# Vortioxetine exhibits anti-glioblastoma activity via the PI3K-Akt signaling pathway

**DOI:** 10.22038/ijbms.2025.82513.17836

**Published:** 2025

**Authors:** Huan-qi Zhang, Dao-ming Zhang, Zhi-zhen Huang, Jing Cheng, Chong Zhang, Neng-ming Lin, Yangling Li

**Affiliations:** 1 Research Center for Clinical Pharmacy, College of Pharmaceutical Sciences, Zhejiang University, Hangzhou 310058, China; 2 Department of Clinical Pharmacology, Key Laboratory of Clinical Cancer Pharmacology and Toxicology Research of Zhejiang Province, Affiliated Hangzhou First People’s Hospital, School of Medicine, Westlake University, Hangzhou 310006, China; 3 School of Medicine, Hangzhou City University, Hangzhou 310015, China

**Keywords:** Akt, Apoptosis, Autophagy, Glioblastoma multiforme, PI3K, Proliferation, Vortioxetine

## Abstract

Glioblastoma multiforme (GBM) presents a significant challenge in oncology due to its highly aggressive nature and inherent resistance to conventional therapeutic interventions. Vortioxetine, a novel antidepressant, exhibits anticancer abilities and can traverse the blood-brain barrier. In this study, the antitumor effect and mechanism of vortioxetine on GBM cells were investigated. Cell proliferation in GBM cells was assessed using the CCK8 and colony formation assays. Flow cytometry, western blot, and wound healing assay were used to study the mechanisms of vortioxetine. mCherry-GFP-LC3B and confocal microscopy were used to evaluate autophagic activity. RNA sequencing uses the capabilities of high-throughput sequencing methods to provide insight into the transcriptome of cells. Vortioxetine significantly inhibited the proliferation of GBM cells by inducing G1/G0 phase cell cycle arrest. Meanwhile, it also reduced the migratory capabilities of GBM cells. Furthermore, it promoted apoptotic cell death in GBM cells. In addition, it promoted autophagy in GBM cells, and autophagy inhibitors markedly enhanced its antiproliferative activities. Vortioxetine could down-regulate the expressions of PI3K and Akt, which were related to the occurrence and development of GBM. Our findings support the potential of vortioxetine as a novel therapeutic agent for GBM treatment. Vortioxetine exhibits anti-GBM activity via the PI3K-Akt signaling pathway. Meanwhile, our findings reveal autophagy inhibitors as an effective sensitizer for vortioxetine, offering new strategies for treating GBM.

## Introduction

Glioblastoma multiforme (GBM) is the most prevalent primary intracranial tumor. It is characterized by a dismal prognosis, with a 5-year mortality rate preceded only by pancreatic and lung cancers among systemic tumors (1). GBM is renowned for its highly aggressive behavior and is marked by its diffuse infiltration into adjacent brain tissue (2). The current standard of care provides limited therapeutic options for patients with GBM and does not substantially improve survival outcomes. Over the past two decades, temozolomide (TMZ) has been the sole drug approved as a first-line treatment for GBM (3). Given TMZ’s limited efficacy and the high incidence of drug resistance, there is a pressing need to explore and develop novel therapeutic strategies to enhance clinical management and treatment outcomes for GBM patients.

The development of effective brain cancer therapies is further complicated by the presence of the blood-brain barrier (BBB), which poses a substantial obstacle to the delivery of therapeutic agents (4). This barrier restricts the access of various chemotherapeutic drugs and immune cells to the central nervous system (5). Nevertheless, accumulating evidence suggests that certain antidepressants, such as fluoxetine, may reduce the incidence of GBM and enhance patients’ quality of life (6). Vortioxetine, classified as a multimodal antidepressant, has shown both efficacy and a favorable safety profile in the management of major depressive disorder (MDD) (7). Functional studies have elucidated that vortioxetine operates primarily through its role as a serotonin transporter (SERT) inhibitor (8). Vortioxetine may offer improved clinical therapeutic outcomes for cancer patients, such as those with gastric cancer (9). Consequently, our research suggests that vortioxetine holds promise as a chemopreventive agent or as an adjuvant therapy for GBM (10).

In this study, we observed that vortioxetine effectively suppresses the progression of GBM cells through modulation of proliferation and apoptosis. Specifically, vortioxetine inhibits the PI3K-Akt signaling pathway, which is known to play a critical role in promoting the progression of GBM. Furthermore, vortioxetine induces cytoprotective autophagy, and the inhibition of autophagy sensitizes vortioxetine’s anti-GBM activity. These findings support the potential of vortioxetine as a novel therapeutic strategy for managing GBM.

## Materials and Methods

### Cell culture

The human malignant glioblastoma cell lines A172, U251, and T98G cell lines were obtained from the Shanghai Institute of Biochemistry and Cell Biology (Shanghai, China). A172 and U251 cells were cultured in high-glucose Dulbecco’s Modified Eagle’s Medium (DMEM) supplemented with 10% FBS (catalog no. FS301-02, TransGen), 100 U/ml penicillin, and 100 μg/ml streptomycin. T98G cells were grown in Minimum Essential Medium (MEM) with the same supplements. All cells were maintained in a humidified incubator at 37 °C with 5% CO_2_ (11).

### Reagents and antibodies

For cell culture, MEM and DMEM were obtained from Hyclone (Logan, UT, USA). Trypsin-EDTA Solution (0.25%) was acquired from Gibco (Grand Island, NY, USA). A pre-stained protein marker was purchased from Thermo Fisher Scientific (Waltham, MA, USA). Vortioxetine was sourced from Aladdin (Shanghai, China) and was dissolved in dimethyl sulfoxide (DMSO) to prepare a stock solution with a concentration of 40 mM. Z-VAD-FMK (catalog no. HY-16658B, MedChemExpress) was also dissolved in DMSO to achieve a stock concentration of 40 mM. 3-Methyladenine (3-MA; catalog no. S2767, Selleck) was prepared in the corresponding culture medium to a final concentration of 50 mM. Chloroquine (CQ; catalog no. S6999, Selleck) was dissolved in DMSO to a stock concentration of 40 mM.

Primary antibodies used in this study included those against Cyclin D1 (catalog no. 2978S; 1:1000), p21 (catalog no. 2947T; 1:1000), β-Tubulin (catalog no. 2128S; 1:1000), N-Cadherin (catalog no. 13116S; 1:1000), Slug (catalog no. 9585S; 1:1000), LC3A/B (catalog no. 12741S; 1:1000), PI3K (catalog no. 4249S; 1:1000), p-mTOR (Ser2448; catalog no. 5536S; 1:1000), mTOR (catalog no. 2983S; 1:1000), p-p70S6K (Thr421/Ser424; catalog no. 9204S; 1:1000), c-Myc (catalog no. 18583S; 1:1000), and cleaved-poly (ADP-ribose) polymerase (cleaved-PARP; catalog no. 9541S; 1:1000), all of which were obtained from Cell Signaling Technology, Inc. The anti-GAPDH antibody (FL-335; catalog no. sc-25778; 1:500) and p-Akt (1/2/3) (Ser473; catalog no. sc-7985; 1:500) antibodies were purchased from Santa Cruz Biotechnology, Inc. The primary antibody against Akt (catalog no. 610836; 1:500) was sourced from BD Biosciences. E-Cadherin (catalog no. ab1416; 1:500), Bim (catalog no. ab32158; 1:500), Bcl-xL (catalog no. ab32370; 1:1000), and p62 (catalog no. ab109012; 1:1000) antibodies were obtained from Abcam Plc.

### Cell viability and proliferation assays

Cells were seeded at a density of 4 × 10³ cells per well in 96-well plates and allowed to adhere overnight. Following adherence, the cells were treated with the specified agents for 72 hr. Cell proliferation was evaluated using the Cell Counting Kit-8 (CCK8) assay (MedChem Express, USA). Specifically, 100 μl of RPMI 1640 medium containing 10 μl of CCK8 solution was added to each well, and the plates were incubated for two hours. Absorbance was measured at 450 nm using a microplate reader (Bio-Rad, USA). Each experiment was conducted in triplicate and repeated independently three times to ensure the reproducibility and reliability of the results (12).

### Colony formation assays

Cells were seeded at a density of 1 × 10³ cells per well in 6-well culture plates and treated with vortioxetine for 14 days. The culture medium containing the specified compounds was refreshed every 3 days. After the incubation period, colonies were stained with 1% crystal violet solution for 30 min. Stained colonies were then washed with tap water, counted, and photographed for subsequent analysis. Colonies containing >50 cells were viewed by light microscope (x40 magnification) and counted using ImageJ software (National Institutes of Health; version v1.8.0) (13).

### Cell cycle distribution analysis

Cells were seeded at a density of 1 × 10⁵ cells per well in a 6-well plate and allowed to adhere overnight. The following day, cells were treated with varying concentrations of vortioxetine (0, 6, 8, 10, 12 μM) for 24 hr. After treatment, cells were harvested, suspended in 250 μl PBS, and fixed in 750 μl of 75% cold ethanol overnight at -20 °C. Cell cycle distribution was assessed using propidium iodide (PI) staining, and 1 × 10⁴ cells per sample were analyzed by flow cytometry (14).

### Cell migration assay

A wound-healing assay was performed to evaluate the migration of A172 and U251 cells treated with vortioxetine. Cells were seeded in six-well plates at a density of 1.5 × 10⁵ cells per well and treated with vortioxetine at concentrations of 2 and 4 μM for 48 hr. Following treatment, a uniform scratch was created across each cell monolayer using a sterile 10-μl pipette tip. Cellular debris was removed by washing with medium, and fresh medium was subsequently added. Images of the scratched areas were captured under light microscopy at 0 hr and 48 hr post-scratching. The results were quantified by calculating the average distance between the edges of the wound to determine the migratory potential of the cells (15).

### Western blot analysis

Cell lysates were prepared with a lysis buffer and centrifuged at 12,000 rpm for 30 min at 4 °C to obtain total proteins. The proteins were separated by SDS-PAGE and transferred onto polyvinylidene fluoride membranes. The membranes were blocked with 5% milk for one hour, then incubated with specific primary antibodies followed by horseradish peroxidase-conjugated secondary antibodies. Protein bands were visualized using an enhanced chemiluminescent substrate and captured on autoradiography film for analysis (16). Each protein band must be subjected to at least three independent replicates to ensure experimental rigor and reproducibility.

### Apoptotic cell death analysis

Apoptosis was assessed using Annexin V/PI staining. Cells were seeded at a density of 5 × 10⁴ cells per well in six-well plates and treated with vortioxetine. After 48 hr of treatment, the cells were stained with the BD Pharmingen™ PE Annexin V Apoptosis Detection Kit I (catalog no. 559763). Apoptotic cells were then analyzed using a FACSCanto™ II flow cytometer (Becton Dickinson) (17).

### Plasmid transfection

Jet-PRIME transfection reagents (Polyplus transfection) were applied to transfect plasmids following the instructions from the manufacturer (18). Cells were transfected with an mCherry-GFP-LC3B plasmid from Loche Biomedicine (China). After 48 hr, cells were treated with vortioxetine for an additional 24 hr. Following treatment, cells were fixed with 3.7% formaldehyde, and the formation of LC3B-positive yellow punctate structures, indicative of autophagosomes, was visualized using a fluorescence microscope. The number of autophagosomes was quantified using ImageJ.

### RNA-sequence

Cells were seeded at a density of 5 × 10⁵ cells per 10-cm culture dish and treated with vortioxetine (8 μM) for 24 hr. After treatment, cells were washed thrice with cold PBS and lysed using Trizol for ten minutes. RNA-containing precipitates were collected and sent to Novogene Bioinformatics Technology (Hangzhou, China) for RNA-sequencing analysis, performed blinded to ensure unbiased result interpretation (19).

### Statistical analysis

Data were presented as mean ± standard deviation (SD). Statistical significance was assessed using a two-tailed Student’s t-test (*P<0.05; **P<0.01; ***P<0.001). Each experiment was conducted independently at least three times to ensure the reproducibility and reliability of the results.

## Results

### Vortioxetine inhibits proliferation and migration in GBM cells

**Figure 1:**
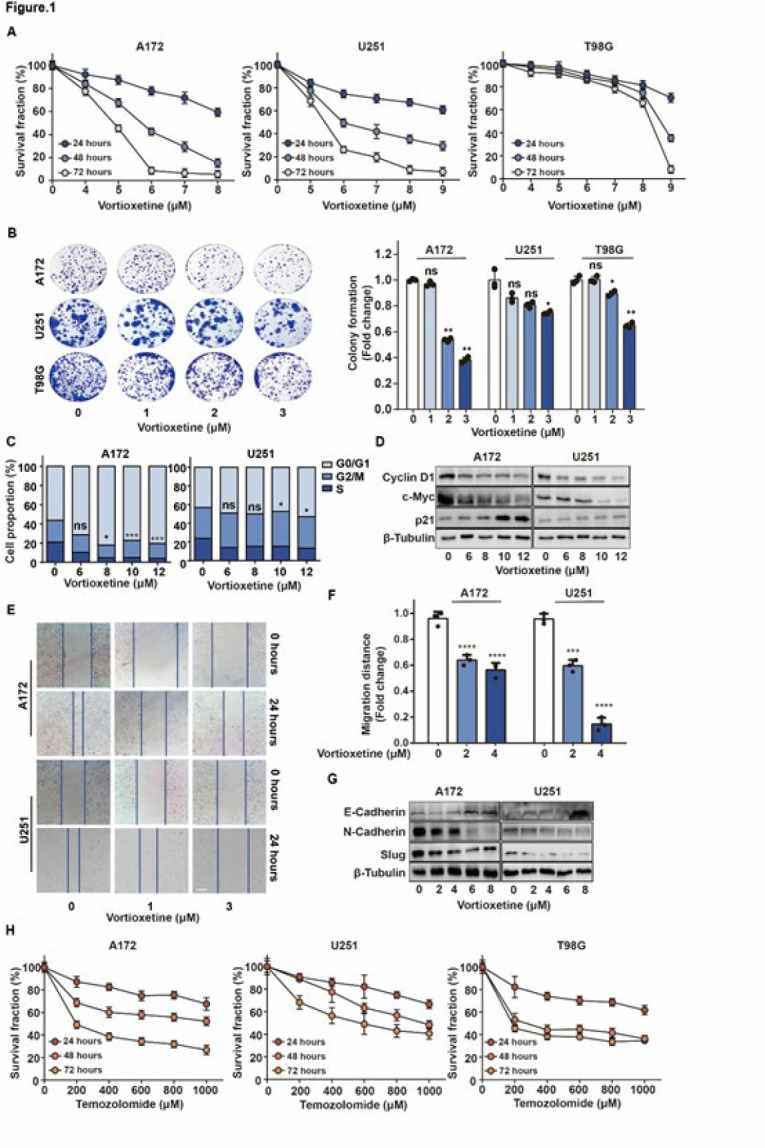
Effects of vortioxetine treatment on cell proliferation, cell cycle distribution, and migration in GBM cells

To investigate the effects of vortioxetine on proliferation in GBM cells, three GBM cells were treated with various concentrations of vortioxetine (from 4 to 9 μM) for 24, 48, and 72 hr. CCK8 analysis revealed that vortioxetine suppressed cell proliferation dose-dependently on A172 and U251 cells. However, the antiproliferative effect of vortioxetine on T98G cells was limited, with the highest IC_50_ among three GBM cells (Figure 1A and Table 1). Meanwhile, vortioxetine treatment showed an antiproliferative effect in a time-dependent manner in three GBM cells (Figure 1A). Subsequently, we investigated the impact of vortioxetine on colony formation of A172, U251, and T98G cells. Treatment with 1 and 3 μM of vortioxetine significantly reduced the number of colonies formation of A172, U251, and T98G cells, respectively (Figure 1B).

Vortioxetine clearly induced cell cycle arrest at the G1/G0 phase in a concentration-dependent manner in GBM cells (Figure 1C). Additionally, we observed that vortioxetine suppressed the levels of Cyclin D1 and c-Myc while up-regulating the level of p21 (Figure 1D). These findings indicate that vortioxetine induces G1/G0 phase cell cycle arrest in GBM cells.

To evaluate the effects of vortioxetine on cell migration, A172 and U251 cells were treated with vortioxetine (2 or 4 μM) for 48 hr. As depicted in Figure 1E, untreated cells effectively migrated to close the wound areas. In contrast, vortioxetine significantly suppressed cell migration to repair a wound (Figure 1F). These results indicate that vortioxetine reduces cell migration in A172 and U251 cells. Furthermore, vortioxetine decreased the levels of N-Cadherin and Slug and increased the level of E-Cadherin (Figure 1G), suggesting that vortioxetine suppresses epithelial-mesenchymal transition (EMT) in GBM cells. Thus, vortioxetine inhibits EMT and the migratory abilities of GBM cells.

To compare with the effects of temozolomide on proliferation in GBM cells, three GBM cells were treated with various concentrations of temozolomide (from 200 to 1000 μM) for 24, 48, and 72 hr (Figure 1H and Table 2). CCK8 analysis revealed that vortioxetine demonstrates a significantly lower IC_50_ than temozolomide, indicating a greater potency in inhibiting GBM cell growth. 

### Vortioxetine induces apoptosis in GBM cells

**Figure 2:**
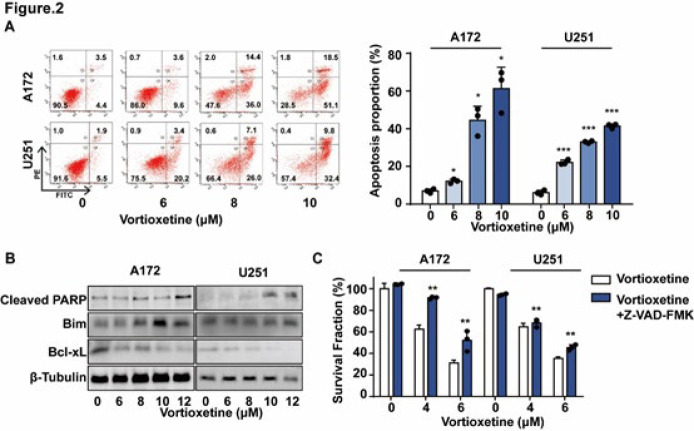
Vortioxetine induces apoptosis in GBM cells

To assess the induction of apoptosis by vortioxetine, we conducted an Annexin V assay in GBM cells. As illustrated in Figure 2A, vortioxetine induced apoptosis in a dose-dependent manner on GBM cells. Additionally, we demonstrated that treatment with vortioxetine significantly induced cleavage of PARP protein, which is crucial for DNA repair and cell death (Figure 2B). Furthermore, vortioxetine decreased the levels of Bcl-xL while increasing the levels of Bim (Figure 2B). Inhibition of programmed cell death by Z-VAD-FMK reversed the anti-proliferation effect of vortioxetine on GBM cells (Figure 2C). These findings collectively indicate that vortioxetine promotes apoptotic cell death in GBM cells.

### Vortioxetine induces autophagy in GBM cells

**Figure 3:**
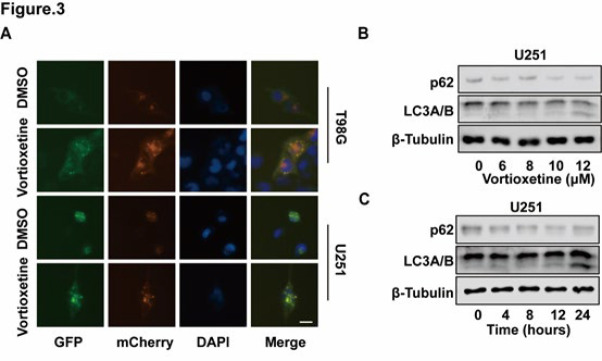
Vortioxetine induces autophagy in GBM cells

In the previous study, vortioxetine induced autophagy in cancer cells (20). Thus, we are interested in investigating whether vortioxetine could induce autophagy in GBM cells. U251 and T98G cells were transfected with mCherry-GFP-LC3B, vortioxetine increased the percentage of GBM cells displaying red and green puncta compared to untreated cells, and vortioxetine induced the formation of autophagosomes, which could be illustrated by yellow puncta, while red puncta signify autolysosomes (Figure 3A). Autophagy is a cellular process involving the interaction between lysosomes and autophagosomes (21). During this process, autophagosomes, which are double-membraned vesicles that sequester cellular components, fuse with lysosomes to form autolysosomes. This fusion results in the degradation of the sequestered materials within the autolysosomes. Furthermore, vortioxetine could induce autophagy via up-regulating autophagy markers such as LC3B and down-regulating p62 (Figure 3B-C). Taken together, these findings strongly indicate that vortioxetine significantly induces autophagy in GBM cells.

### Inhibition of autophagy potentiates vortioxetine-induced cytotoxicity

**Figure 4:**
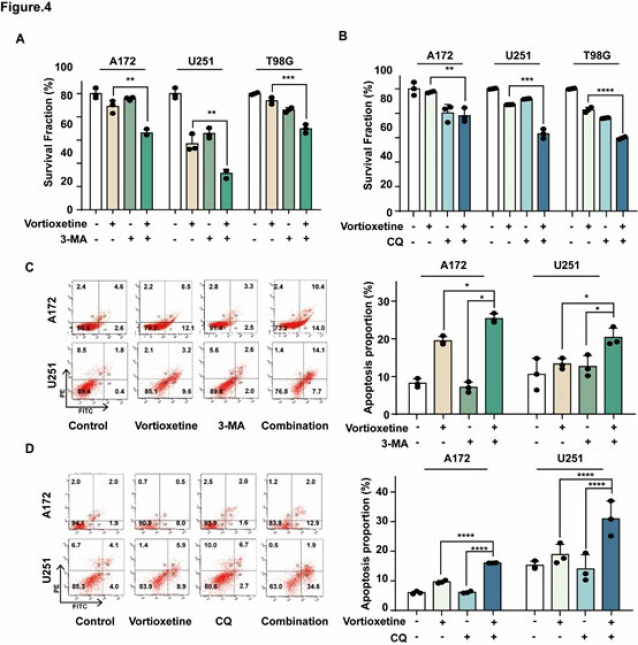
Autophagy inhibition enhances vortioxetine-induced apoptosis in GBM cells

3-MA and CQ are known as typical autophagy inhibitors, and flow cytometric analysis demonstrated that the suppression of autophagy using autophagy inhibitor treatment could enhance vortioxetine-induced apoptosis in GBM cells (Figure 4C-D). Furthermore, the combination of vortioxetine with 3-MA or CQ markedly reduced cell viability compared to vortioxetine treatment alone (Figure 4A-B). These findings were corroborated by western blot analysis, revealing that 3-MA and CQ effectively suppressed vortioxetine-induced accumulation of LC3B. At the same time, co-treatment with vortioxetine and 3-MA/CQ substantially increased PARP cleavage compared to vortioxetine treatment alone (Figure 4E-F). These results suggest that vortioxetine-induced autophagy may exert a protective effect against apoptosis in GBM cells.

### Vortioxetine suppresses the activation of the PI3K-Akt pathway

**Figure 5:**
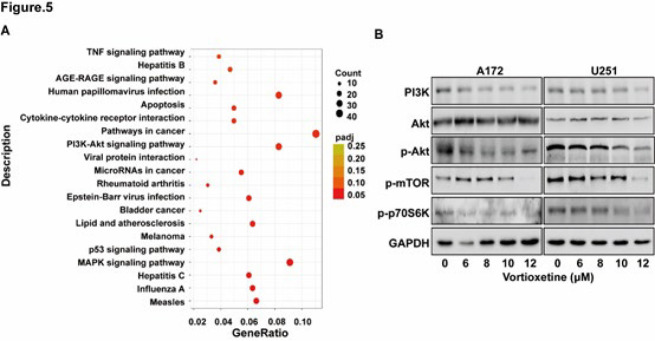
Vortioxetine inhibits activation of the PI3K-Akt signaling pathway

To elucidate the molecular mechanism of vortioxetine in GBM, we conducted a functional enrichment analysis of key proteins regulated by vortioxetine using RNA-sequence followed by STRING. Vortioxetine was found to potentially modulate several signaling pathways, including PI3K-Akt, MAPK, p53, AGE-RAGE, and TNF pathways (Figure 4A). PI3K-Akt and MAPK signaling pathways were highly enriched. The PI3K-Akt pathway is crucial for regulating cellular processes such as growth, survival, autophagy, and apoptosis (22). Further, PI3K induces the activation of AKT and downstream activation of other protein kinases, such as MAPK (23). Then, we detected whether vortioxetine could regulate the PI3K pathway in GBM cells. As depicted in Figure 5B, vortioxetine treatment significantly reduced the levels of PI3K and p-Akt compared to the negative control group. These results demonstrate that vortioxetine effectively inhibits the PI3K-Akt signaling pathway in GBM cells.

## Discussion

GBM, the most common and aggressive primary brain tumor, is characterized by a high mortality rate and displays significant intra- and intertumor heterogeneity, which contributes to treatment resistance and eventual tumor recurrence (24). Complete surgical resection is challenging due to the tumor’s extensive local infiltration and distant metastases throughout the brain (25-28). Currently, the approved therapeutic agents for GBM treatment include TMZ, carmustine, and bevacizumab. Other therapeutic agents have shown limited success in clinical trials, mainly due to their difficulty in crossing the blood-brain barrier, a well-known challenge in brain cancer treatment, and the inherent resistance of the highly interconnected glioblastoma tumors (29). Advancements in the treatment of GBM are crucial due to the tumor’s aggressive nature and resistance to conventional therapies (30). Therapeutic agents targeting the central nervous system (CNS), such as antidepressants, are being explored for their potential repurposing in the treatment of GBM. Recently, certain antidepressants, including vortioxetine, have demonstrated antiproliferative activity in cancer cells. Vortioxetine, as a CNS-targeted therapeutic agent capable of crossing the BBB, presents a novel approach for GBM treatment. Recent studies have shown that vortioxetine exhibits promising anti-GBM activity (31). 

Several studies indicate that inhibiting the PI3K-Akt-MAPK signaling pathway demonstrates anti-GBM effects (32). This underscores an urgent need for the development of safe and effective PI3K inhibitors, and it is important to assess whether a PI3K inhibitor has sufficient brain penetration prior to starting its development for the treatment of intracranial cancers. Vortioxetine, notable as the first antidepressant, operates through a mechanism that involves the inhibition of serotonin reuptake along with the modulation of various SERTs (33). In some instances, SERT antagonists have proven effective in inhibiting cancer cell growth, with this effect partially mediated through modulation of the PI3K-Akt signaling pathway (34-36). Thus, vortioxetine may exert antitumor effects against GBM as a PI3K inhibitor. 

Autophagy, a conserved cellular mechanism crucial for maintaining homeostasis and energy supply, has become an important process that limits antitumor activity (37). The Akt protein is downstream of the PI3K protein and activates other protein kinases, such as MAPK (23). The traditional role of Akt in autophagy is antagonistic. Thus, we hypothesize that vortioxetine may induce autophagy via PI3K in GBM cells. Moreover, autophagy might be a protective symptom. Thus, autophagy inhibitors potentially sensitize tumor cells to vortioxetine and could serve as adjunctive treatments for GBM (38, 39).

Cells were treated with increasing concentrations of vortioxetine, and cell proliferation was assessed at 24, 48, and 72 hr using the CCK8 assay (A). Colony formation assays were performed in A172, U251, and T98G cells treated with vortioxetine to determine colony formation capacity (B). Cell cycle distribution in A172 and U251 cells treated with vortioxetine for 24 hr was evaluated by PI staining (C). Western blot analysis was conducted to determine the expression levels of specific proteins in cells treated with vortioxetine for 24 hr (D). Cells treated with increasing concentrations of vortioxetine were evaluated for migration capacity at 48 hr in A172 and U251 cells (E-F). Western blot analysis of indicated protein expression after 24 hr of treatment with vortioxetine or DMSO control (G). Cells were treated with increasing concentrations of temozolomide, and cell proliferation was assessed at 24, 48, and 72 hr using the CCK8 assay (H). The scale bar indicated a length of 50 μm. Statistical significance was denoted as ns (non-significant), *P<0.05, **P<0.01, ***P<0.001, and ****P<0.0001.

CCK8: Cell Counting Kit-8; DMSO: Dimethyl sulfoxide; PI: Propidium iodide

**Table 1:** IC_50_ of vortioxetine in GBM cells

A172		U251		T98G	
Time (hr)	IC_50 _(μM)	Time (hr)	IC_50 _(μM)	Time (hr)	IC_50 _(μM)
24	9.18 ± 0.80	24	11.41 ± 2.05	24	11.45 ± 1.02
48	5.73 ± 0.18	48	6.53 ± 0.83	48	8.62 ± 0.61
72	4.78 ± 0.31	72	5.45 ± 0.48	72	8.10 ± 0.66

IC_50_: Half-maximal inhibitory concentrations; GBM: Glioblastoma multiforme

**Table 2:** IC_50_ of temozolomide in GBM cells

A172		U251		T98G	
Time (hr)	IC_50 _(μM)	Time (hr)	IC_50 _(μM)	Time (hr)	IC_50 _(μM)
24	3388 ± 9	24	2066 ± 4	24	2897 ± 8
48	1590 ± 5	48	1016 ± 9	48	271 ± 1
72	168 ± 3	72	570 ± 5	72	103 ± 4

IC_50_: Half-maximal inhibitory concentrations; GBM: Glioblastoma multiforme

Cells were treated with increasing concentrations of vortioxetine for 48 hr and subsequently stained with Annexin V and PI. Positively stained cells were quantified using BD FACSCanto^TM^II, with a representative flow plot (A). Cells were treated with vortioxetine or DMSO for 48 hr, and western blot analysis was performed to determine the expression levels of specific proteins (B). Cell viability assays were conducted in A172 and U251 cells co-cultured with indicated programmed cell death inhibitors under various doses of vortioxetine for 72 hr (C). Z-VAD-FMK (10 μM), an apoptosis inhibitor. Statistical significance was denoted as ns (non-significant), *P<0.05, **P<0.01, ***P<0.001, and ****P<0.0001.

DMSO: Dimethyl sulfoxide; PI: Propidium iodide

U251 and T98G cells were transfected with mCherry-GFP-LC3B plasmids and treated with or without vortioxetine. Representative images of mCherry-GFP-LC3B-labeled autophagosomes, with scale bars indicating 10 μm (A). U251 cells were treated with increasing concentrations of vortioxetine (0, 6, 8, 10, or 12 μM), and autophagy-related proteins were assessed by western blot analysis (B). U251 cells treated with vortioxetine (8 μM) were evaluated for changes in autophagy-related protein expression at 4, 8, 12, and 24 hr using western blot analysis (C). The scale bar indicated a length of 10 μm.

GBM: Glioblastoma multiforme

A172, U251, and T98G cells were treated with vortioxetine alone or in combination with 3-MA for 72 hr. Cell viability was assessed using the CCK8 assay (A). A172, U251, and T98G cells were treated with vortioxetine alone or in combination with CQ for 72 hr. Cell viability was determined using the CCK8 assay (B). Apoptosis was quantified by flow cytometric analysis (C-D).

GBM: Glioblastoma multiforme; CCK8: Cell Counting Kit-8; CQ: Chloroquine

DEGs regulated by vortioxetine were analyzed for functional enrichment using the STRING database (A). A172 and U251 cells were treated with increasing concentrations of vortioxetine. Western blot analysis was performed to assess the expression levels of PI3K/Akt pathway proteins (B).

DEGs: Differentially expressed genes

## Conclusion

In summary, this study demonstrates that vortioxetine induces apoptosis while simultaneously eliciting an autophagic response by inhibiting the PI3K-Akt signaling pathway in GBM cells. Notably, suppressing this autophagic process enhances vortioxetine-induced apoptosis, indicating it is a protective autophagic response. These results clarify the anti-GBM effect and mechanism of vortioxetine and suggest that combining vortioxetine with autophagy inhibitors may represent a novel chemotherapeutic strategy for treating GBM.

## Data Availability

**The data presented in the study are included in the article and additional material.**

## Declaration

**We acknowledge the use of ChatGPT 3.5 to enhance language quality and check for grammatical errors. After using this tool, we reviewed and edited the content as needed and take full responsibility for the content of the publication.**

## References

[1] Hisaoka K, Nishida A, Takebayashi M, Koda T, Yamawaki S, Nakata Y (2004). Serotonin increases glial cell line-derived neutrophic factor release in rat C6 glioblastoma cells. Brain Res.

[2] Bi J, Khan A, Tang J, Armando AM, Wu S, Zhang W (2021). Targeting glioblastoma signaling and metabolism with a repurposed brain-penetrant drug. Cell Rep.

[3] Song T, Li H, Tian Z, Xu C, Liu J, Guo Y (2015). Disruption of NF-kappaB signaling by fluoxetine attenuates MGMT expression in glioma cells. OncoTargets Ther.

[4] Steingart AB, Cotterchio M (1995). Do antidepressants cause, promote, or inhibit cancers. J Clin Epidemiol.

[5] Siddiqui EJ, Thompson CS, Mikhailidis DP, Mumtaz FH (2005). The role of serotonin in tumour growth. Oncol Rep.

[6] Wang Y, Wang X, Wang X, Wu D, Qi J, Zhang Y (2021). Imipramine impedes glioma progression by inhibiting YAP as a Hippo pathway independent manner and synergizes with temozolomide. J Cell Mol Med.

[7] Zhou J, Xu N, Liu B, Wang C, He Z, Lenahan C (2022). lncRNA XLOC013218 promotes cell proliferation and TMZ resistance by targeting the PIK3R2-mediated PI3K/AKT pathway in glioma. Cancer Sci.

[8] Dizeyi N, Hedlund P, Bjartell A, Tinzl M, Austild-Taskén K, Abrahamsson PA (2011). Serotonin activates MAP kinase and PI3K/Akt signaling pathways in prostate cancer cell lines. Urol Oncol.

[9] Li M, Duan L, Wu W, Li W, Zhao L, Li A (2023). Vortioxetine hydrobromide inhibits the growth of gastric cancer cells in vivo and in vitro by targeting JAK2 and SRC. Oncogenesis.

[10] Schmidt L, Kling T, Monsefi N, Olsson M, Hansson C, Baskaran S (2013). Comparative drug pair screening across multiple glioblastoma cell lines reveals novel drug-drug interactions. Neuro Oncol.

[11] Yao T, Asayama Y (2017). Animal-cell culture media: History, characteristics, and current issues. Reprod Med Biol.

[12] Cai L, Qin X, Xu Z, Song Y, Jiang H, Wu Y (2019). Comparison of cytotoxicity evaluation of anticancer drugs between real-time cell analysis and CCK-8 method. ACS Omega.

[13] Franken N, Rodermond H, Stap J, Haveman J, Bree C (2006). Clonogenic assay of cells in vitro. Nat Protoc.

[14] Kim H, Kim J, Yu S, Lee YY, Park J, Choi RJ (2020). A Mechanism for microRNA arm switching regulated by uridylation. Mol Cell.

[15] Pijuan J, Barceló C, Moreno DF, Maiques O, Sisó P, Marti RM (2019). In vitro cell migration, invasion, and adhesion assays: From cell imaging to data analysis. Front Cell Dev Biol.

[16] Edfors F, Hober A, Linderbäck K, Maddalo G, Azimi A, Sivertsson Å (2018). Enhanced validation of antibodies for research applications. Nat Commun.

[17] Hollville E, Martin SJ (2016). Measuring apoptosis by microscopy and flow cytometry. Curr Protoc Immunol.

[18] Lin X, Liu YH, Zhang HQ, Wu LW, Li Q, Deng J (2023). DSCC1 interacts with HSP90AB1 and promotes the progression of lung adenocarcinoma via regulating ER stress. Cancer Cell Int.

[19] Koch CM, Chiu SF, Akbarpour M, Bharat A, Ridge KM, Bartom ET (2018). A Beginner’s guide to analysis of rna sequencing data. Am J Respir Cell Mol Biol.

[20] Ryskalin L, Limanaqi F, Biagioni F, Frati A, Esposito V, Calierno MT (2017). The emerging role of m-TOR up-regulation in brain Astrocytoma. Histol Histopathol.

[21] Behrends C, Sowa ME, Gygi SP, Harper JW (2010). Network organization of the human autophagy system. Nature.

[22] Tewari D, Patni P, Bishayee A, Sah AN, Bishayee A (2022). Natural products targeting the PI3K-Akt-mTOR signaling pathway in cancer: A novel therapeutic strategy. Semin Cancer Biol.

[23] Morgos DT, Stefani C, Miricescu D, Greabu M, Stanciu S, Nica S (2024). Targeting PI3K/AKT/mTOR and MAPK signaling pathways in gastric cancer. Int J Mol Sci.

[24] Merzak A, Koochekpour S, Fillion MP, Fillion G, Pilkington GJ (1996). Expression of serotonin receptors in human fetal astrocytes and glioma cell lines: A possible role in glioma cell proliferation and migration. Brain Res Mol Brain Res.

[25] Cheer SM, Goa KL (2001). Fluoxetine: A review of its therapeutic potential in the treatment of depression associated with physical illness. Drugs.

[26] Hosseinimehr SJ, Najafi SH, Shafiee F, Hassanzadeh S, Farzipour S, Ghasemi A (2020). Fluoxetine as an antidepressant medicine improves the effects of ionizing radiation for the treatment of glioma. J Bioenerg Biomembr.

[27] Sarrouilhe D, Clarhaut J, Defamie N, Mesnil M (2015). Serotonin and cancer: What is the link?. Curr Mol Med.

[28] Meyer N, Henkel L, Linder B, Zielke S, Tascher G, Trautmann S (2021). Autophagy activation, lipotoxicity and lysosomal membrane permeabilization synergize to promote pimozide- and loperamide-induced glioma cell death. Autophagy.

[29] Yin H, Lu H, Yang JH, Li Q, Li M, Zhao Q (2024). Daurisoline suppresses glioma progression by inhibiting autophagy through PI3K/AKT/mTOR pathway and increases TMZ sensitivity. Biochem Pharmacol.

[30] Morita K, Arimochi H, Itoh H, Her S (2006). Possible involvement of 5alpha-reduced neurosteroids in adrenergic and serotonergic stimulation of GFAP gene expression in rat C6 glioma cells. Brain Res.

[31] Devare MN, Kaeberlein M (2024). An antidepressant drug vortioxetine suppresses malignant glioblastoma cell growth. MicroPubl Biol.

[32] D’Alessandro G, Lauro C, Quaglio D, Ghirga F, Botta B, Trettel F (2021). Neuro-signals from gut microbiota: Perspectives for brain glioma. Cancers.

[33] Zifa E, Fillion G (1992). 5-Hydroxytryptamine receptors. Pharmacol Rev.

[34] Karpel-Massler G, Kast RE, Westhoff MA, Dwucet A, Welscher N, Nonnenmacher L (2015). Olanzapine inhibits proliferation, migration and anchorage-independent growth in human glioblastoma cell lines and enhances temozolomide’s antiproliferative effect. J Neurooncol.

[35] Hsu FT, Chiang IT, Wang WS (2020). Induction of apoptosis through extrinsic/intrinsic pathways and suppression of ERK/NF-kappaB signalling participate in anti-glioblastoma of imipramine. J Cell Mol Med.

[36] Balakrishna P, George S, Hatoum H, Mukherjee S (2021). Serotonin pathway in cancer. Int J Mol Sci.

[37] Sorice M (2022). Crosstalk of Autophagy and Apoptosis. Cells.

[38] Lv GB, Wang TT, Zhu HL, Wang HK, Sun W, Zhao LF (2020). Vortioxetine induces apoptosis and autophagy of gastric cancer AGS cells via the PI3K/AKT pathway. FEBS Open Bio.

[39] Seitz C, Hugle M, Cristofanon S, Tchoghandjian A, Fulda S (2013). The dual PI3K/mTOR inhibitor NVP-BEZ235 and chloroquine synergize to trigger apoptosis via mitochondrial-lysosomal cross-talk. Int J Cancer.

