# Supplementary material for: Vortioxetine exhibits anti-glioblastoma activity via the PI3K-Akt signaling pathway
Source: Iran J Basic Med Sci. 2025;28(4):401–8. doi: 10.22038/ijbms.2025.82513.17836 (PMC11831746; doi:10.22038/ijbms.2025.82513.17836)
Supplement: Supplementary file 1 [file ijbms-28-401-s001.pdf]

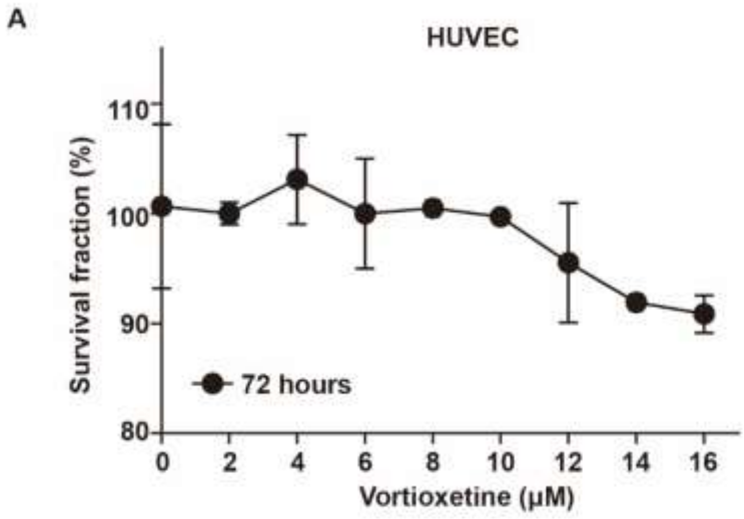

Fig S1. Effects of vortioxetine treatment on cell proliferation in non-cancerous cells

HUVEC stands for Human Umbilical Vein Endothelial Cells, which are endothelial cells extracted from the lining of the human umbilical vein within the umbilical cord. HUVEC Cells were treated with increasing concentrations of vortioxetine, and cell proliferation was assessed at 72 hours using the CCK8 assay.

Table S1. IC_{50} of vortioxetine in HUVEC cells

HUVEC	
Time (hr)	IC_{50} (μM)
72	30.11 ± 1.31
